# Case of recurring Kikuchi disease and autoimmune hepatitis

**DOI:** 10.1002/ccr3.6459

**Published:** 2022-10-17

**Authors:** Umaima Dhamrah, Branden Ireifej, Sibghatallah Ummar, Nuzhat Batool, David Song, Nirali Sheth, Abhigan Babu Shrestha, Vikash Jaiswal

**Affiliations:** ^1^ Department of Internal Medicine NYC Health & Hospitals/Elmhurst Hospital Center Elmhurst New York USA; ^2^ Department of Medicine Bahria University Medical & Dental College Karachi Pakistan; ^3^ Department of Medicine M Abdur Rahim Medical College Dinajpur Bangladesh; ^4^ Larkin Community Hospital South Miami Florida USA

**Keywords:** autoimmune hepatitis, kikichi disease, steroids

## Abstract

We present a case of a 47‐year‐old female patient with a history of diagnosed Kikuchi disease and autoimmune hepatitis 13 years ago who presented with recurrent fevers and a desquamative rash on the lower extremities. Computed tomography neck showed enlarged lymph nodes, and with her daily fevers and skin rashes the presentation was concerning for recurrence of her Kikuchi disease. The patient was also found to have an elevated anti‐smooth muscle antibody titer, and subsequent liver biopsy confirmed the diagnosis of autoimmune hepatitis. She was started on methylprednisolone with improvement. Our case emphasizes the association of Kikuchi disease with autoimmune conditions other than systemic lupus erythematosus. Given the recurrence of the disease after a decade of quiescence, long‐term follow‐up of patients with Kikuchi disease should be implemented.

## Abstract 2

Kikuchi disease (KD) can relapse despite a remission period, with other recurrent autoimmune diseases closely linked. Our case emphasizes the association of KD with autoimmune conditions other than systemic lupus erythematosus (SLE). Given the recurrence of the disease after a decade of quiescence, long‐term follow‐up of patients with KD should be implemented.

## 1 BACKGROUND

Kikuchi's disease (KD) is a rare, benign disease that is characterized by fever and localized lymphadenopathy, and its association with autoimmune diseases has been reported. KD has been reported throughout literature with a close connection to SLE,^1^, ^2^ among other autoimmune diseases. We report a case of recurring KD, originally in remission for several years, along with a concomitant flare up of biopsy proven autoimmune hepatitis (AIH).

## 2 CASE PRESENTATION

A 47‐year‐old Hispanic female patient with a history of KD diagnosed 13 years ago presented with recurrent fevers, desquamative rash on the legs, and diffuse arthralgias. She reported daily fevers previously. One week prior to her presentation, she developed abdominal pain associated with nausea, anorexia, and dark urine. Vital signs on arrival were unremarkable without evidence of hypotension, tachycardia, or hypoxia. Physical examination was significant for a desquamative rash on her thighs bilaterally with no clinical lymphadenopathy. Infectious work up including chest X‐ray, urinalysis, complete blood count, and blood cultures were unremarkable. Further laboratory results revealed an elevated AST 241 units/L, ALT 277 units/L, and ALP 118 units/L (normal 3 months earlier). Autoimmune work up only showed weakly positive ASMA with titers of 1:40 while ANA, anti‐dsDNA, anti‐SSA/SSB were negative. Ultrasound of the abdomen showed cholelithiasis without cholecystitis or hepatic steatosis. CT neck showed a 1.8 cm right submandibular, 1.6 cm left submandibular, subcentimeter submental and supraclavicular lymph nodes. Her lymphadenopathy with daily fevers, skin rash, and joint pain was concerning for recurrence of her KD. Given her elevated liver function tests, there was also concern for concomitant acute AIH. Patient then underwent liver biopsy which confirmed AIH. Patient was subsequently started on IV methylprednisolone 1 mg/kg with improvement in her liver function tests along with her fevers and arthralgias. She was started on azathioprine 50 mg PO at discharge and continued to follow‐up in rheumatology and liver clinic as outpatient for management of her disease. She began to have symptomatic improvement after about 4–6 months on the medication.

**FIGURE 1:**
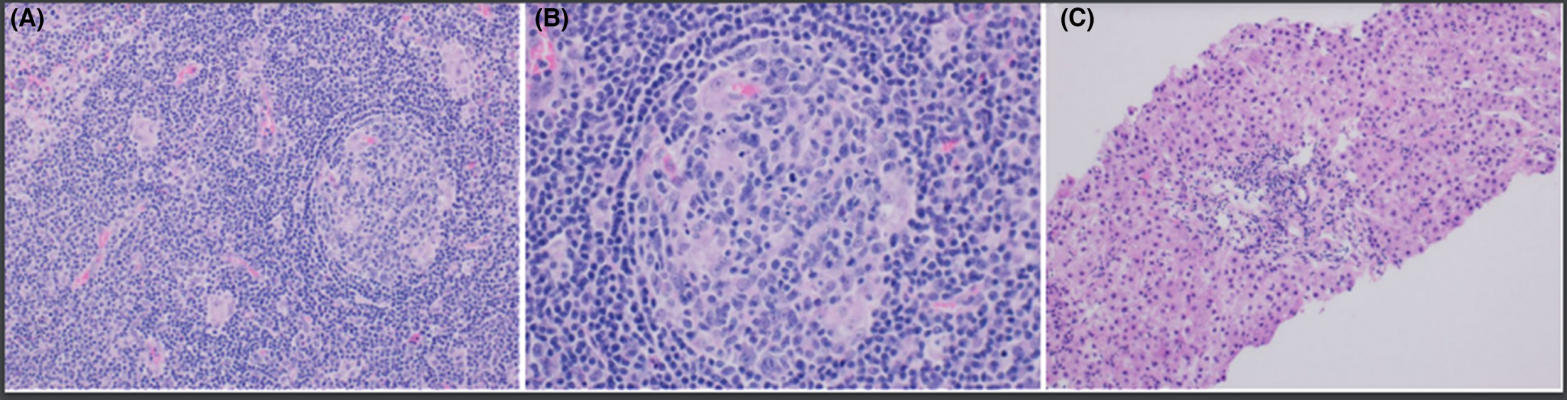
(A,B): Axillary lymph node biopsy showing cortical and paracortical areas of necrosis with lymphocyte phagocytosis shown on the stain, many histolytic collections with some plasma cells, and eosinophils. (C): Liver biopsy showing plasma cells and fibrosis, consistent with autoimmune hepatitis

The patient had a known history of AIH associated with KD, which was diagnosed 13 years ago via liver biopsy and axillary lymph node biopsy, respectively (Figure 1). Work up at the time of diagnosis was negative for ANA, anti‐dsDNA, anti‐Sm, anti‐RNP and positive for elevated liver enzymes. She was started on corticosteroids and azathioprine, which was self‐discontinued after 8 years. Her symptoms only began to reappear after 1–2 years off medication.

## 3 DISCUSSION

The extranodal symptoms of KD are uncommon and diverse including skin rash, night sweats, weight loss, headache, cough, and abdominal pain.^3^ The disease resolves spontaneously in weeks to months, but in some cases, it may have systemic involvement or evolve to SLE.^1^, ^2^, ^4^, ^5^, ^6^ A definite diagnosis of KD is based on characteristic pathologic findings on biopsy that differentiate this disease from others such as lymphoma, and infectious lymphadenopathy. Characteristic histopathologic findings include necrotic and thrombotic blood vessels. The karyorrhectic foci are formed by different cellular types, predominantly histiocytes and plasmacytoid monocytes, but also immunoblasts and small and large lymphocytes.^7^

The recurrence of KD is rare. There have been more recent reports of recurrence rates as high as 13% in an Asian population in Korea.^8^ In a study by Jung et al, 54 patients (11.3%) experienced 1–4 recurrent episodes of KD each. The initial recurrence occurred within a mean duration of 6 months (range: 1 month to 6 years). Patients with recurrent KD were more likely to have extranodal symptoms and lymphopenia. Although the etiology of KD recurrence is unknown, certain viral infections have been hypothesized to be among the triggers for KD relapse.^9^

Patients with KD showing progression to autoimmune diseases were more likely to have fever, common extranodal symptoms, a higher recurrence rate, and a higher ANA positivity rate at KD diagnosis.^3^ Hepatosplenomegaly was present in 18% of patients of KD^9^ with few cases having clinically diagnosed AIH. Our case had biopsy proven AIH without clinical and laboratory findings of SLE. In addition, our case had recurrence of symptoms of KD after 9 years which improved after starting corticosteroids.

## 4 CONCLUSION

KD is a rare condition, and it should be considered in differential diagnoses of tender lymphadenopathy, especially lymphadenopathy localized to the cervical region and recurrent fever with AIH. Although the disease takes a self‐limiting clinical course in most cases, we reported a case of KD with a prolonged relapse of 10 years. Our case emphasizes the association of KD with autoimmune conditions other than SLE. Full recovery with a good response to corticosteroid regimen was achieved after the recurrence; therefore, considering the recurrence of KD, long‐term follow‐up of patients with KD should be implemented.

## AUTHOR CONTRIBUTIONS

UD, BI, NU, and DS were involved in patient care (diagnosis, treatment, and follow‐up). UD, BI, NU, NS, VJ, AS, and SU contributed to the collection of case information, writing of the manuscript, and manuscript revision. VJ and DS were involved in revising the manuscript critically for important intellectual content. All authors approved the final version.

## FUNDING INFORMATION

The authors have no financial support to disclose.

## CONFLICT OF INTEREST

The authors have no conflicts of interest to disclose.

## ETHICAL APPROVAL

Institutional Review Board (IRB) approval is not required for case reports at our institution.

## CONSENT

Written informed consent was obtained by a patient. A copy of the written consent is available for review by the Editor‐in‐Chief of this journal.

## Data Availability

The data used in the case report are available on reasonable request.
